# A Comprehensive Review of Current and Emerging Treatments for Narcolepsy Type 1

**DOI:** 10.3390/jcm14238444

**Published:** 2025-11-28

**Authors:** Qinglin Xu, Yigang Chen, Tiantian Wang, Qiongbin Zhu, Jiahui Xu, Lisan Zhang

**Affiliations:** 1 Department of Neurology, Sir Run Run Shaw Hospital, School of Medicine, Zhejiang University, Hangzhou 310016, China; 2 Department of Pharmacy, Xiasha Campus, Sir Run Run Shaw Hospital, School of Medicine, Zhejiang University, Hangzhou 310009, China

**Keywords:** narcolepsy type 1, cataplexy, excessive daytime sleepiness, orexin receptor agonist, pharmacological treatment, orexin system

## Abstract

Narcolepsy Type 1 (NT1) is a rare chronic neurological disorder characterized by core clinical manifestations such as excessive daytime sleepiness (EDS), cataplexy, sleep paralysis (SP), hypnagogic and hypnopompic hallucinations (HHs), and disrupted nocturnal sleep (DNS). Patients often experience comorbidities, including cognitive impairment, psychiatric disorders, and metabolic syndrome, necessitating lifelong management. Current therapeutic approaches primarily involve pharmacologic treatments for symptomatic relief, supplemented by non-pharmacologic interventions aimed at alleviating EDS and cataplexy. However, existing therapies are limited in efficacy and do not offer a cure. In recent years, a deeper understanding of the central role played by the orexin (hypocretin) system in the pathogenesis of NT1 has led to breakthrough advances in mechanism-based therapies targeting this pathway. Notably, selective orexin-2 receptor (OX2R) agonists such as TAK-861 have shown remarkable efficacy in Phase II/III clinical trials, holding the potential to fundamentally reshape the NT1 treatment landscape. This review systematically outlines current treatment options for NT1, with a focus on management strategies for atypical symptoms and special populations. It also highlights emerging therapeutic directions—including orexin-targeted agents, immunotherapies, and orexin cell/gene treatments—along with their future development.

## 1. Introduction

Narcolepsy is a disabling chronic neurological disorder with a prevalence of approximately 1 in 2000 [1]. According to the International Classification of Sleep Disorders, 3rd Edition (ICSD-3), the disorder is primarily classified into Type 1 (NT1) and Type 2 (NT2). NT1 is characterized by cataplexy and significantly reduced orexin levels in cerebrospinal fluid (<110 pg/mL) [1]. Its pathogenesis is closely associated with genetic susceptibility (e.g., HLA-DQB1*06:02 allele), autoimmune mechanisms (e.g., orexin-specific CD4+ and CD8+ T cell-mediated neuronal damage), and environmental triggers (e.g., H1N1 influenza infection or vaccination) [2]. The core pathological alteration involves defects in the hypothalamic orexin system, characterized by the specific loss or epigenetic silencing of orexin-producing neurons [3]. Patients typically exhibit low cerebrospinal fluid (CSF) orexin (Hcrt-1) levels, leading to disruption of the sleep–wake regulatory circuitry [4].

The classic “pentad” of NT1 symptoms includes excessive daytime sleepiness (EDS), cataplexy, disrupted nocturnal sleep (DNS), sleep paralysis (SP), and hypnagogic/hypnopompic hallucinations (HH) [3]. However, its clinical manifestations extend far beyond this scope, often accompanied by significant cognitive impairment, psychiatric symptoms (such as depression and anxiety), and autonomic dysfunction. It frequently coexists with comorbidities such as obesity, cardiovascular disease, and sleep apnea, severely impacting patients’ quality of life and social functioning [5]. Current treatment strategies for NT1 primarily focus on symptom control, improving daytime functioning, and preventing complications. Although existing medications (such as wakefulness-promoting agents, sodium oxybate, antidepressants, etc.) relieve symptoms, they cannot reverse the disease progression. They also present challenges, including side effects, insufficient efficacy, and significant interindividual variability. Therefore, exploring novel therapies based on disease mechanisms is crucial.

This review outlines individualized management for narcolepsy type 1 (NT1), including pharmacological and non-pharmacological strategies, and critically evaluates emerging therapies such as orexin-targeted drugs, immunotherapies, and cell/gene-based treatments. Through integration of current evidence and recent advances, it aims to guide clinical practice and inform the development of next-generation NT1 therapies.

## 2. Current Treatment

NT1 exhibits high heterogeneity in severity [6]. While primarily characterized by excessive daytime sleepiness and cataplexy, it is frequently accompanied by multiple comorbidities [7,8]. Management should be individualized, integrating pharmacological and non-pharmacological strategies tailored to individual profiles. Initial monotherapy with agents targeting multiple symptoms is recommended. Special attention is warranted for specific populations (e.g., children, women of childbearing potential, the elderly). Close follow-up with regimen adjustments based on efficacy and feedback is essential.

### 2.1. Pharmacological Treatment

Pharmacological treatment is the cornerstone for managing the core symptoms of narcolepsy type 1 (NT1). Internationally recognized guidelines—specifically those from the American Academy of Sleep Medicine (AASM) [9] and the European guidelines on narcolepsy management [10]—offer evidence-based recommendations for both adult and pediatric populations, though they differ in certain details and scope. Current pharmacological treatments based on these two guidelines are summarized in Table 1 and Table 2, while the pharmacology of narcolepsy treatments is outlined in Table 3 and Table 4.

#### 2.1.1. EDS Pharmacological Treatment

##### Modafinil

Modafinil, a non-amphetamine stimulant, acts by (1) inhibiting dopamine transporters (DAT) to increase dopamine; (2) activating norepinephrine α1B receptors to promote norepinephrine release; (3) increasing glutamate and decreasing GABA, enhancing excitatory neurotransmission; and (4) reducing oxidative stress via glutathione elevation [11]. RCTs show modafinil reduces ESS scores by 4–6 points (*p* < 0.001), prolongs MWT sleep latency by 3–5 min (*p* < 0.001), and improves fatigue (30–40% reduction), mood (20–25% reduction), and cognitive function (15–20% improvement) [12,13]. A 12-month study demonstrated sustained EDS improvement in 80–85% of patients with no significant tolerance [14]. It has no major effects on adult sleep, but in children, it may prolong sleep onset latency (SOL) (*p* = 0.014), requiring morning dosing [15]. Adult dose: start at 100 mg/day, increase to 200–400 mg/day if needed. Pediatric: 50–400 mg/day (2–8 mg/kg) once daily [16]. Common side effects: headache, insomnia, and nausea, usually resolving in 1–2 weeks. Serious rare effects: psychiatric symptoms (e.g., hallucinations, <1%), Stevens-Johnson syndrome [17,18,19]. Pregnancy: EMA/ANSM alert for potential congenital malformation risk early in pregnancy, but studies conflict; risk unconfirmed. Recommend discontinuing use in early pregnancy [19,20,21].

##### Pitolisant

Pitolisant, a selective H3-receptor competitive antagonist/inverse agonist, acts via blocking autoreceptors to enhance histamine release, moderately modulating dopamine/norepinephrine systems, and inhibiting GABAergic neuronal activity [22]. In the Harmony-CTP trial, the 36 mg/day dose significantly reduced ESS scores by 5–7 points (*p* < 0.001), prolonged MWT by 4–6 min (*p* < 0.001), and decreased weekly cataplexy episodes by 75% (*p* < 0.001) [23]. Real-world studies demonstrated sustained efficacy, with ESS scores declining from 15.3 to 10.5, alongside improved quality of life (EQ-5D-5L VAS +12.3) and reduced depressive symptoms (BDI from 7.5 to 4.7) [24,25]. Pediatric trials (≥6 years) showed a 6.3-point reduction in UNS total score (*p* = 0.007), decreased ESS from 19 to 13.5 (*p* < 0.001), and reduced cataplexy frequency [26,27]. Pharmacokinetics: ~90% oral absorption, ~20-h half-life, CYP2D6-metabolized, no dose adjustment for hepatic/renal impairment [22]. Dosing: adults start at 9 mg/day, increasing to 18–36 mg/day after 2 weeks; children start at 4.5 mg/day, increasing to 9–36 mg/day [10]. Common adverse events include headache, insomnia, nausea, and anxiety, mostly mild and transient. Serious adverse events were rare (0.5%), with long-term safety confirmed [25]. Minimal cardiovascular impact makes it suitable for elderly patients. A drug-holiday regimen reduced modafinil dosage by 41% (*p* < 0.0001) while improving side effects [10,28].

##### Solriamfetol

Solriamfetol is a wake-promoting agent that inhibits dopamine and norepinephrine transporters (DAT/NET) [29]. It is indicated for excessive daytime sleepiness (EDS) in narcolepsy and obstructive sleep apnea (OSA), especially in narcolepsy type 1 with comorbid OSA. The drug is rapidly absorbed (Tmax ~2 h) and has a half-life of ~7.1 h, with ~90% excreted unchanged in urine. Dose adjustment is required for renal impairment: reduce by half for eGFR 30–59 mL/min/1.73 m^2^ and avoid if eGFR < 15 [30].

Randomized controlled trials have shown improvements in EDS. In a 12-week Phase III trial, solriamfetol (150 mg and 300 mg) increased MWT duration and reduced ESS scores, irrespective of cataplexy status [31]. Another Phase III trial reported mean MWT increases of 9.8 and 12.3 min (vs. 2.1 for placebo), and ESS reductions of 5.4 and 6.4 points (vs. 1.6 for placebo) for the 150 mg and 300 mg doses, respectively [32]. A meta-analysis of six RCTs showed a mean MWT increase of 9.93 min and ESS reduction of 4.44 points, alongside higher improvement rates on Patient and Clinician Global Impression of Change scales (PGI-C/CGI-C) [29]. Network meta-analyses have reported that solriamfetol was ranked as superior to pitolisant, sodium oxybate, and modafinil/armodafinil in improving ESS and MWT [33,34]. Real-world data (SURWEY) supported these findings, with ESS scores decreasing from 17.6 to 13.6 (mean reduction: 4.3 points) [35].

Safety profiles are generally favorable. Common adverse events include headache, nausea, and decreased appetite, with incidence rates similar to placebo in clinical trials [35]. Solriamfetol has low abuse potential (<1% drug craving) and no withdrawal rebound [29]. Mild increases in blood pressure (~2–3 mmHg systolic) and heart rate (~2–3 bpm) may occur but usually require no intervention [30]. Efficacy and safety are unaffected by depression history [36]. During long-term treatment (52 weeks), 25.7% of patients experienced ≥5% weight loss, showing a dose-dependent pattern [37]

##### Sodium Oxybate (SXB) and Its Derivatives

Sodium Oxybate (SXB, brand name Xyrem^®^), the sodium salt of gamma-hydroxybutyric acid (GHB), is the only agent that simultaneously improves EDS, cataplexy, and disrupted nighttime sleep (DNS) in narcolepsy. It modulates neurotransmission by binding to GHB receptors and activating GABAB receptors, improving sleep architecture by increasing slow-wave sleep and reducing nocturnal awakenings [37]. It exhibits rapid absorption and a short half-life, necessitating twice-nightly dosing on an empty stomach [38]. The adult starting dose is 4.5 g/night, titratable to a maximum of 9 g/night [16,39].

Clinical trials confirm SXB’s efficacy. It reduces weekly cataplexy attacks in a dose-dependent manner [40] and significantly improves EDS, with additive effects when combined with modafinil [41]. SXB also enhances sleep continuity and quality [42,43]. A systematic review of 9 RCTs confirmed its benefits for EDS, cataplexy, and sleep structure [44]. Common adverse reactions are nausea, dizziness, and headache [45], though psychiatric symptoms have been reported [46,47]. Long-term use is associated with weight loss and a significant sodium load (1100–1640 mg/night), equating to 2.8–4.2 g of salt intake nightly, posing potential cardiovascular risks [48,49]. A network meta-analysis indicated that LXB had the most favorable tolerability profile among anti-EDS drugs [34].

Low-Sodium Oxybate (LXB, brand name Xywav^®^)

LXB is a mixture of oxybate salts with 92% less sodium than SXB. It is indicated for patients requiring sodium restriction [50]. Phase 3 trials demonstrate non-inferior efficacy to SXB in reducing EDS and cataplexy, with a reduced risk of cardiovascular adverse events [51]. Long-term use is associated with weight loss and improved quality of life [52].

Once-Nightly Sodium Oxybate (ON-SXB, brand name Lumyrz^®^)

ON-SXB is an extended-release formulation allowing single bedtime dosing, improving adherence compared to SXB [53]. The Phase 3 REST-ON trial confirmed its efficacy in improving EDS, sleep quality, and cataplexy within weeks of treatment [54]. Its safety profile is similar to SXB, with no new signals identified [54].

Clinical Caution Box

Sodium Burden of Oxybate Formulations: The high sodium burden associated with SXB (sodium oxybate) may induce hypertension, thereby increasing cardiovascular risk. However, a formal cardiovascular risk model quantifying the long-term benefits of LXB (low-sodium oxybate) over SXB is currently lacking. Thus, conducting relevant pharmacoepidemiological research to fill this evidence gap is crucial.

##### Other Wake-Promoting Agents

Armodafinil: The active R-enantiomer of modafinil features a longer half-life (12–15 h) for once-daily use. It shows efficacy comparable to modafinil for EDS, but with a lower incidence of insomnia (10% vs. 15%) [55]. Long-term use may cause mild blood pressure increases and tachycardia, necessitating regular monitoring [56].

Methylphenidate: A potent dopamine reuptake inhibitor with significant stimulant effects and high abuse potential. It improves EDS at doses of 10–60 mg/day but is ineffective for cataplexy, making it a choice for refractory cases only [57]. Common adverse effects include tachycardia, hypertension, and insomnia, with a 5–10% risk of abuse/dependence [58].

Amphetamines: These agents exert the strongest wake-promoting effects by enhancing dopamine/norepinephrine release and reuptake inhibition. While effective for refractory EDS, they are ineffective for cataplexy and carry the highest risks of serious adverse effects—including arrhythmias, psychosis, and dependence—and are contraindicated in cardiovascular disease [10,16].

#### 2.1.2. Cataplexy Pharmacological Treatment

Anti-cataplexy drugs act primarily by modulating serotonin and norepinephrine to prevent emotion-induced loss of muscle tone. First-line treatments include sodium oxybate (SXB) and pitolisant.

##### First-Line Treatment

SXB activates GABAB receptors, inhibiting brainstem motor inhibitory pathways and stabilizing REM sleep to reduce cataplexy [59]. Its benefits include dose-dependent efficacy and absence of withdrawal rebound upon discontinuation [60]. Pitolisant promotes histamine release, enhancing brainstem arousal and moderately modulating dopamine, thereby reducing cataplexy frequency [61]. Advantages are convenient once-daily dosing and no negative cognitive effects, making it suitable for individuals requiring sustained mental performance [23].

##### Second-Line Treatment (Antidepressants)

Antidepressants, including venlafaxine, clomipramine, fluoxetine, and citalopram, inhibit serotonin/norepinephrine reuptake to increase sympathetic tone. They are reserved for patients unresponsive to first-line agents. European guidelines offer a weak recommendation, while AASM guidelines do not endorse them due to limited evidence. A key advantage is their concurrent benefit for comorbid depression/anxiety.

Venlafaxine (an SNRI) at 37.5–225 mg/day in adults reduces cataplexy by 50–60% [62]. Common adverse effects are hypertension, headache, and dry mouth. Abrupt withdrawal may trigger cataplectic status, necessitating gradual tapering [63]. Clomipramine (a TCA), at 10–75 mg/day, is highly effective (60–70% reduction) but limited by anticholinergic effects like dry mouth, tachycardia, and risk of hypotension [64,65,66]. SSRIs (e.g., fluoxetine, citalopram) exhibit weaker efficacy (30–40% reduction) and are reserved for mild-to-moderate cases; side effects include agitation, nausea, and sexual dysfunction [67].

##### Exacerbation of Cataplexy Following Withdrawal of Antidepressants

Discontinuation of antidepressants can provoke cataplexy rebound. A 2009 study showed a significant increase in weekly attacks after stopping TCAs (median 55.7) or SSRIs (median 35.7) versus controls (15.25), which normalized after 4 weeks [68]. Gradual tapering is essential to mitigate this risk.

### 2.2. Nonpharmacological Treatment

Patients with narcolepsy often experience significant psychosocial impairment and reduced quality of life, including diminished work productivity, sexual dysfunction, higher traffic accident risks, and increased neuropsychiatric comorbidities like depression and anxiety [69,70,71]. Pharmacological treatment alone is frequently inadequate. Non-pharmacological therapies, which are safe and sustainable, enhance drug efficacy, reduce dosage, and improve long-term prognosis. European guidelines strongly recommend these interventions as cornerstone treatments for NT1 [10], such as cognitive behavioral therapy, psychological support, sleep hygiene, and lifestyle management [16].

Cognitive behavioral therapy (CBT) is a core non-pharmacological intervention increasingly used in narcolepsy [72]. Its goals include: (1) Cognitive restructuring to modify dysfunctional beliefs and stigma; (2) Behavioral adjustment to improve adherence, sleep hygiene, and scheduled napping; (3) Emotion and stress management, using techniques like systematic desensitization for cataplexy [16]. A tailored CBT for hypersomnia (CBT-H) demonstrated significant ESS score reductions and 15–20% improvements in SF-36 physical and social functioning among 35 patients [73].

Specific behavioral strategies include:

Scheduled napping: Planning 2–3 short naps per day, each lasting 15–20 min, can help reduce sleep inertia and improve daytime alertness. The duration should be adjusted based on individual differences [74,75].

Extending nocturnal sleep duration: When total sleep time increases from 417.0 min to approximately 505.2 min, the daytime sleep latency of patients with narcolepsy significantly increased (*p* < 0.01) and the number of naps with a sleep latency of less than 10 min decreased (*p* < 0.02), and subjective sleepiness scores decreased (*p* < 0.02), indicating that extending night-time sleep can help patients with narcolepsy to reduce EDS [76].

Physical activity: Regular exercise can stabilize circadian rhythms and improve sleep quality. Studies have shown that physical activity is associated with lower subjective sleepiness scores and reduced daytime napping in children with narcolepsy [77].

Environmental and behavioral modulation: Strategies such as controlling ambient temperature [78], avoiding monotonous activities, increasing social interaction, and employing self-stimulating behaviors (e.g., pinching the skin, chewing gum) can temporarily enhance alertness.

Dietary modifications: The low-carbohydrate ketogenic diet (LCKD) is currently the most evidence-supported approach. One study reported an 18% reduction in the total Narcolepsy Symptom Status Questionnaire (NSSQ) score (161.9 vs. 133.5, *p* = 0.0019), with subscale scores decreasing by 22% for sleepiness, 13% for sleep attacks, and 24% for sleep paralysis. Urinary ketone levels peaked at two weeks (3.7 ± 0.9), correlating with symptom improvement, suggesting a potential role of ketone metabolism in the therapeutic mechanism [79]

For cataplexy, CBT utilizes systematic desensitization through gradual exposure to emotional triggers (e.g., laughter) alongside relaxation training to mitigate symptom responses. Additional strategies include emotional avoidance and stimulus satiation [16,80]. Psychological counseling aids patients and families in comprehending disease impact, formulating individualized coping methods, and improving treatment adherence. Peer support alleviates isolation, bolsters coping confidence, and offers practical guidance [16]. Social adjustments—such as extended test time and flexible work schedules—along with peer groups and patient organizations, enhance life quality.

Tailored interventions are essential: pediatric care involves school support with scheduled naps and prolonged test durations, with 83% of parents noting academic improvement [81]. Reproductive-aged women benefit from non-pharmacological approaches coordinating sleep with contraception or pregnancy [82]. Older adults, often with comorbidities, require low-risk measures like consistent sleep–wake routines and sleep education, while avoiding prolonged daytime naps [83,84]. Although resources are limited, online platforms present new opportunities for patient connectivity.

In summary, non-pharmacological treatments, especially CBT and behavioral strategies, significantly improve psychosocial function and daytime symptoms in narcolepsy (Table 5).

### 2.3. Special Populations

Pediatric Patients: Pharmacological treatment in children requires careful consideration of growth, development, and potential long-term effects. Safety signals of particular concern include insomnia and delayed sleep onset (especially with modafinil), cardiovascular effects such as tachycardia and hypertension (with stimulants), and anticholinergic side effects (with tricyclic antidepressants). Dose adjustments are primarily based on body weight (e.g., modafinil, SXB). Data on the QTc interval effects and orthostatic hypotension for many drugs in this population are insufficient. There is a pressing need for more stratified studies focusing on the long-term safety and efficacy of narcolepsy treatments in children [10,16].

Geriatric Patients: Older adults with narcolepsy often present with multiple comorbidities and polypharmacy, increasing the risk of drug interactions and adverse events. Key considerations include the anticholinergic load (from TCAs), which can exacerbate cognitive impairment or cause urinary retention; orthostatic hypotension (from TCAs, venlafaxine); and QTc prolongation risk (associated with several antidepressants and pitolisant). Sodium oxybate use requires caution due to its CNS depressant effects and high sodium load in patients with heart failure or hypertension. Dose initiation should follow a “start low and go slow” principle. However, robust clinical trial data for most narcolepsy medications in the elderly are lacking, representing a significant evidence gap [10,16].

## 3. Emerging Treatments

### 3.1. Orexin System Targeted Therapy

It is well established that type 1 narcolepsy involves defects in the hypothalamic orexin system, characterized by specific loss or epigenetic silencing of orexin-producing neurons in the lateral hypothalamus, resulting in markedly reduced or absent cerebrospinal fluid orexin. Orexin, a key wake-promoting neuropeptide, acts on two G protein-coupled receptors (OX1R and OX2R) and projects widely to multiple arousal-regulatory brain regions, thereby maintaining and stabilizing wakefulness. In narcolepsy type 1, loss of orexin signaling leads to two core symptoms: excessive daytime sleepiness due to inadequate arousal drive and REM sleep dysregulation, where elements such as muscle atonia intrude into wakefulness, causing cataplexy, sleep paralysis, and hypnagogic hallucinations. Thus, compensating for deficient orexin signaling represents a fundamental therapeutic approach, primarily through either orexin replacement or receptor agonism. Given orexin peptides have poor blood-brain barrier permeability and short half-lives, receptor agonists—particularly selective OX2R agonists, which are crucial for promoting wakefulness—offer greater clinical potential. By precisely activating the arousal pathway, OX2R agonists may fundamentally alleviate core narcolepsy symptoms, shifting treatment from symptomatic to pathophysiology-targeted therapy.

#### 3.1.1. Orexin Receptor Agonists

Research has focused on small-molecule receptor agonists. Among these, OX2R selective agonists have emerged as a research hotspot due to their advantages in improving core symptoms and safety profiles. Relevant preclinical and clinical trials have confirmed their therapeutic potential.

##### Preclinical Studies of OX2R Selective Agonists

Early preclinical studies established the therapeutic potential of OX2R-selective agonists. In 2017, Yoko Irukayama-Tomobe et al. demonstrated that the novel non-peptide agonist YNT-185 exerts pharmacological activity in transfection, brain slice, and mouse models, supporting a mechanistic treatment for narcolepsy-cataplexy [85]. A 2022 study in orexin knockout mice showed that the selective OX2R agonist [Ala^11^, D-Leu^15^]-orexin-B (AL-OXB) and non-selective orexin-A both alleviated core symptoms, with AL-OXB exhibiting a superior safety profile, confirming OX2R agonism is sufficient for efficacy [86]. Further studies on danavorexton (TAK-925) revealed rapid binding kinetics (dissociation t_1_/_2_ 0.87 ± 0.10 min) and an activation profile similar to native orexin. In narcolepsy mouse models, it promoted wakefulness, reduced sleep fragmentation, and induced no significant receptor desensitization [87]. TAK-925 and ARN-776, despite low brain penetration, dose-dependently delayed NREM sleep onset, reduced cataplexy, and increased gamma power in EEG [88].

In 2023, the orally available OX2R agonist TAK-994 activated human OX2R (EC_50_ 19 nM) with >700-fold selectivity and improved sleep fragmentation and cataplexy in mouse models, showing stable efficacy after chronic dosing [89]. The 2024 discovery of TAK-861 (oveporexton) marked a breakthrough: it activated OX2R with an EC_50_ of 2.5 nM and ∼3000-fold selectivity over OX1R. It promoted wakefulness in mice and monkeys with ∼10-fold higher potency than TAK-994 and significantly improved symptoms in narcolepsy models without inducing tolerance [90]. Additionally, phenylglycine-based OX2R agonists 57 and 58, identified via high-throughput screening, showed nanomolar potency (EC_50_ = 2.5 and 0.4 nM, respectively) and favorable brain penetration after optimization [91].

##### Clinical Trials of OX2R Selective Agonists

Multiple clinical trials have validated the efficacy of OX2R selective agonists in human patients while providing direction for drug optimization.

TAK-925: Two 2022 clinical studies of danavorexton (TAK-925-1001 single-dose escalation, TAK-925-1003 multiple-dose escalation) demonstrated that intravenous infusion of this drug produced a dose-dependent prolongation of the Multiple Sleep Latency Test (MSLT) sleep latency in NT1 patients (with a maximum effect of up to 40 min), significantly improved EDS and cataplexy. NT2 patients also showed EDS improvement, with overall good tolerability and no serious adverse events [92].

TAK-994: In 2023, the phase 2 randomized placebo-controlled trial and extension study of TAK-994 enrolled 73 patients. Results showed that all dose groups (30mg, 90mg, 180mg twice daily) demonstrated significant improvements in: Epworth Sleepiness Scale (ESS) scores (difference from placebo: −10.1 to −13.0 points), and weekly cataplexy attack rates (incidence ratio 0.05–0.20). However, the trial was terminated early due to 5 cases of significant liver enzyme elevation and 3 cases meeting Hy’s law criteria for drug-induced liver injury (DILI). Subsequent mechanistic studies revealed the liver injury was covalent binding-dependent idiosyncratic DILI, not directly linked to OX2R, providing a target for future drug optimization [93,94].

TAK-861: A Phase 2 trial published in 2025 enrolled 112 patients (90 receiving different doses of TAK-861 and 22 receiving a placebo). After 8 weeks of treatment, all dose groups demonstrated a mean increase in MWT sleep latency of 13.7–26.6 min compared to placebo (adjusted *p* ≤ 0.001) and a reduction in ESS total scores of 6.4–11.3 points compared to placebo (adjusted *p* < 0.005). The weekly cataplexy rate was significantly lower in the 2 mg twice daily and 2 mg followed by 5 mg daily groups compared to placebo (adjusted *p* < 0.05), with no hepatotoxicity observed. 97% of patients completed the trial [95]. Recently, data from two Phase III clinical trials of TAK-861 (The First Light and The Radiant Light) presented at the 2025 World Sleep Congress further confirmed its efficacy. Regarding improvement in daytime sleepiness: In the First Light study, 83% (53/64) of patients in the 2 mg/2 mg dose group achieved an ESS score ≤ 10 at Week 12, compared to only 17% (2/12) in the placebo group. In The Radiant Light study, these proportions were 84% (56/67) and 12% (4/33), respectively. For enhancing daytime alertness, the proportion of patients in the 2mg/2mg dose group with MWT sleep latency ≥ 20 min was 54% (36/67) in The First Light study, compared to 9% (2/22) in the placebo group. In the Radiant Light study, this proportion reached 80% (46/57) compared to 0% in the placebo group. Regarding reduction in cataplexy episodes, the median number of cataplexy-free days at baseline was 0 days for all patients; In the First Light study, the median number of days without cataplexy at Week 12 increased to approximately 4 days in the 1 mg/1 mg and 2 mg/2 mg dose groups, compared to 0.5 days in the placebo group, with weekly cataplexy rate (WCR) reductions of 66% and 79% from baseline, respectively. In the Radiant Light study, the 2 mg/2 mg dose group showed a median drop in cataplexy days to 5 days, with a 79.0% reduction in WCR [96,97].

Furthermore, OX2R agonists show potential in mood regulation. Lihua Chen et al. reported that repeated intraperitoneal injections of the OX2R agonist YNT-185 reduced both baseline and morphine withdrawal-induced anxiety-like behaviors in male mice, suggesting OX2R as a potential therapeutic target for anxiety disorders [98].

Other investigational drugs such as ALKS 2680 and ORX750 are currently in various clinical development stages, demonstrating active progress in this field. Table 6 lists all OX2R agonists currently in clinical trials.

#### 3.1.2. Orexin Replacement Therapy

Orexin replacement therapy compensates for endogenous deficiency by exogenously supplementing orexin peptides. Both animal experiments and human studies demonstrate its efficacy in improving certain symptoms. However, its core limitations lie in the difficulty of crossing the blood-brain barrier and its poor therapeutic effect on EDS.

##### Efficacy and Dosage Exploration in Animal Studies

A 2000 study showed that systemic administration of orexin-A (3 μg/kg) increased activity, prolonged wakefulness, decreased REM sleep without altering non-REM sleep, reduced sleep fragmentation, and produced a dose-dependent reduction in cataplexy in canine narcoleptics. Repeated daily dosing consolidated wake and sleep periods and abolished cataplexy for over three days post-treatment [99]. However, subsequent work revealed that orexin-A replacement was ineffective in dogs with Hcrtr2 mutations. Only very high intravenous doses (96–384 µg/kg) transiently reduced cataplexy, underscoring the blood-brain barrier (BBB) limitation [100]. Later advances explored alternative delivery routes. In 2007, both intravenous (2.5–10.0 μg/kg) and intranasal (1.0 μg/kg) Orexin-A improved cognitive performance in sleep-deprived monkeys, with intranasal administration being more effective than the highest IV dose [101]. A 2008 rat study confirmed that intranasal delivery yielded much lower plasma levels but approximately 80% of the brain concentration—Area under the time curve (AUC) resulted from direct nasal-to-brain transport, confirming intranasal administration bypasses the blood-brain barrier and reduces systemic exposure [102]. A study found that continuous intrathecal delivery of Orexin-A (1 nmol/1 μL/h) ascends from the spinal cord to the brain, elevating intracerebral orexin levels to endogenous levels in wild-type mice. This significantly reduced cataplexy episodes and sleep-onset rapid eye movement (SOREM) episodes, with receptor-specific efficacy [103].

##### The Findings and Limitations of Human Clinical Trials

A series of randomized controlled trials (RCTs) by Baier et al. identified therapeutic effects of intranasal Orexin A in narcolepsy with cataplexy patients. One study confirmed mild olfactory dysfunction as an inherent symptom linked to central orexin deficiency, reversible by intranasal Orexin A [104]. A pilot study of 8 patients showed that 435 nmol intranasal human recombinant Orexin-A before bedtime did not affect nocturnal arousals but reduced REM sleep duration (*p* = 0.02), total REM sleep duration (*p* = 0.039), and direct wakefulness-to-REM transitions [105]. Another trial demonstrated stabilized REM sleep and improved attention with intranasal Orexin A [106]. Current evidence indicates intranasal Orexin A reduces wakefulness-to-REM transitions and total REM duration, improves attention, but does not increase wakefulness or alleviate sleepiness [104,105,106].

### 3.2. Immunotherapy

Although no direct evidence currently confirms that narcolepsy type 1 (NT1) is an autoimmune disease, increasing indirect evidence supports the autoimmune hypothesis:

- Genetic Susceptibility: Narcolepsy shows a strong association with HLA-DQB1*06:02 [107], a molecule responsible for antigen presentation to CD4+ T cells. It is also associated with polymorphisms in immune-related genes such as TCR-α and P2RY11 [108,109].
- Environmental Triggers: 1. Streptococcal Infection: Individuals who developed streptococcal pharyngitis before age 21 exhibit increased NT1 risk, with higher serum streptococcal antibody levels within 3 years of onset compared to controls [110]; 2. H1N1 infection/vaccination: NT1 incidence tripled within 6 months following China’s H1N1 pandemic [111]; increased incidence among Pandemrix vaccine recipients [112,113], suggesting molecular mimicry between the H1N1 HA protein and hypocretin [114].
- Immune cell involvement: 1. CD4+ T cells: Patients harbor hypocretin-specific CD4+ T cells capable of cross-recognizing H1N1 HA protein [114]; 2. CD8+ T cells: Increased autoreactive CD8+ T cells in patient blood [115], and in animal models, CD8+ T cells can directly destroy orexin neurons [116]; CD8+ T cell clones in CSF correlate with progression from NT2 to NT1 [117].
- Evidence of autoantibodies: 1. TRIB2 antibody: Positive in 14% of patients, but also positive in 5% of controls. Acts as an intracellular antigen with no direct pathogenic role [118]; 2. HCRTR2 antibody: Positive in 85% of post-vaccination NT1 cases (vs. 35% controls), absent in idiopathic NT1, lacking core pathogenic significance [119].

Based on the autoimmune hypothesis, various immunotherapy regimens have been attempted for patients with narcolepsy, including corticosteroids, plasma exchange (PLEX), intravenous immunoglobulin (IVIG), and rituximab. However, most reports are case studies or short-term applications with inconsistent efficacy—for example, IVIG improves cataplexy only in some patients with recent-onset disease, while PLEX provides only temporary relief. Long-term effects remain unvalidated by randomized controlled trials. Recently, targeted therapies against B cells and T cells have also been explored in limited case studies. Table 7 summarizes various immunotherapies in NT1 [120,121,122,123,124,125,126,127,128].

### 3.3. Cell Therapy and Gene Therapy

As a long-term vision for fundamental treatment, these strategies aim to restore the function of the orexin system.

#### 3.3.1. Cell Transplantation

The core pathogenesis of narcolepsy involves massive loss of hypothalamic orexin neurons, leading to significantly reduced cerebrospinal fluid orexin levels. Existing treatments only partially alleviate symptoms and cannot reverse neuronal loss. Successful transplantation of orexin neurons could potentially serve as a fundamental therapeutic approach. Transplantation experience in Parkinson’s disease demonstrates that fetal dopaminergic neurons can survive for over 10 years post-transplantation, integrate into host circuits, and restore function, providing feasibility evidence for orexin neuron transplantation [129,130]. Experimental evidence has shown that the suspension of orexin neurons extracted from the hypothalamus of 8–10-day-old rat pups, when transplanted into the pons of adult rats (the area where orexin fibers project, without their own neurons), can survive until 36 days after transplantation. The morphology of the surviving neurons is similar to that of mature orexin neurons in adult rats, but the number continuously decreases starting from day 3. This preliminary verification demonstrates the feasibility of orexin neuron cell transplantation [131].

#### 3.3.2. Gene Transfer

In recent years, orexin gene supplementation therapy has gained significant research interest. Several studies have successfully improved symptoms in narcoleptic mouse models by delivering the orexin gene to specific brain regions via viral vectors, laying the groundwork for clinical translation. Liu et al. first used a replication-defective HSV-1 amplicon vector to deliver the mouse prepro-orexin gene into the lateral hypothalamus (LH) of orexin knockout mice. Over a 4-day expression period, cataplexy decreased by ~60%, REM sleep duration in the latter half of the night normalized to wild-type levels, and orexin-A was detected in the CSF, confirming the efficacy of targeted gene transfer [132].

The same group employed a recombinant AAV (rAAV) vector in orexin-ataxin-3 transgenic mice. They found that orexin delivery to the zona incerta (ZI) or LH significantly reduced cataplexy without altering total sleep–wake time, whereas striatal delivery or targeting MCH neurons was ineffective, underscoring regional specificity. Tracer studies indicated that ZI receives amygdala inputs and projects to the locus coeruleus, suggesting its role in stabilizing motor tone [133]. Blanco-Centurion et al. injected rAAV-orexin into the dorsolateral pons of orexin KO mice, reporting an 80.7% reduction in cataplexy and an increased proportion of long wake bouts at night (8% to 23%), indicating improved wake maintenance. Notably, control GFP injection worsened cataplexy, highlighting regional sensitivity [134].

Kantor et al. targeted the mediobasal hypothalamus in Atx transgenic mice. Following AAV-orexin injection, wake time increased by 13% and wake bout duration lengthened by 48% during the dark phase, with improved diurnal patterns of wakefulness and REM sleep, though cataplexy was not significantly reduced, suggesting symptom-specific neural pathways [135]. Liu et al. further investigated the amygdala’s role by delivering rAAV-orexin into its central and basolateral nuclei in orexin KO mice. This not only reduced spontaneous cataplexy but also blocked odor-induced cataplexy and increased wakefulness during odor exposure, providing the first evidence that orexin gene transfer suppresses both spontaneous and emotion-triggered cataplexy [136].

**Figure 1:**
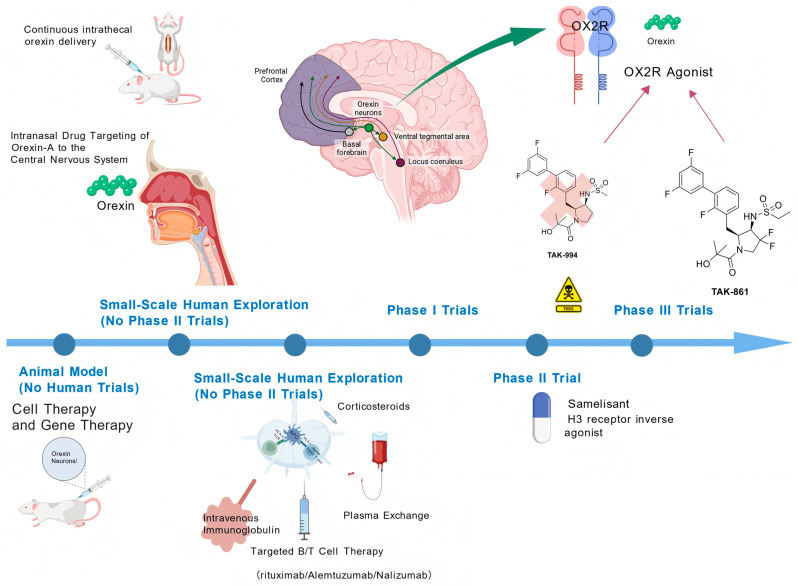
Emerging Treatment for Narcolepsy Type 1.

Figure 1 shows the merging Treatment for Narcolepsy Type 1.

This illustration outlines key therapeutic strategies targeting the orexin (hypocretin) system—a core pathogenic pathway in narcolepsy Type 1 (NT1)—alongside other emerging treatments. Key elements include the following: Orexin Receptor Agonists: Selective orexin-2 receptor (OX2R) agonists (e.g., TAK-861, TAK-994) and other orexin agonists, which directly activate wake-promoting pathways via central nervous system (CNS) targets such as the prefrontal cortex, ventral tegmental area, and locus coeruleus. Orexin Replacement Therapy: Delivery approaches for orexin-A (e.g., intranasal, continuous intrathecal administration) to bypass the blood-brain barrier and compensate for endogenous orexin deficiency. Emerging Treatments: Immunotherapies (intravenous immunoglobulin, plasma exchange, targeted B/T cell therapies including rituximab, alemtuzumab, nalizumab), cell/gene therapies, corticosteroids, and histamine H3 receptor inverse agonists (e.g., samelisant). Clinical Development Phases: Classification of agents by developmental status (animal models, small-scale human exploration, Phase I/II/III trials) to highlight translational progress. This visualization synthesizes mechanism-based interventions and novel therapeutic directions reshaping NT1 management. Created with BioGDP.com

### 3.4. Other New Drugs

Samelisant (SUVN-G3031) is a novel, potent, selective histamine H3 receptor (H3R) inverse agonist currently under development for the treatment of narcolepsy. It exhibits favorable ADME properties, including high passive permeability, high plasma free fraction, and primarily renal excretion (accounting for ~78% of total human clearance). Its metabolism relies on CYP3A4 and MAO-A, posing minimal risk of drug interactions. Preclinical studies indicate it modulates neurotransmitter levels such as histamine and dopamine, prolongs wakefulness duration, and reduces cataplexy episodes. Phase 2 clinical trials confirmed significant improvement in excessive daytime sleepiness (with marked reduction in ESS scores), while common adverse reactions include insomnia and abnormal dreams. A sensitive LC-MS/MS quantitative method has been established for clinical detection, and related research is advancing it toward later clinical phases [137].

## 4. Discussion

Currently, the clinical management of narcolepsy type 1 (NT1) primarily relies on symptomatic treatment. Although these drugs can prolong wakefulness to some extent, their ability to improve the quality remains limited. In recent years, selective OX2R agonists have emerged as a novel mechanism-driven therapeutic strategy for NT1. By activating downstream arousal pathways of OX2R, these agonists not only simultaneously improve EDS, cataplexy, and nocturnal sleep fragmentation, but also enhance overall wakefulness quality—including heightened attention and reduced anxiety. The representative drug TAK-861 was evaluated in Phase II clinical trials and was associated with restoration of arousal function to levels considered healthy in most patients. After 8 weeks of treatment, the proportion of patients achieving a mean sleep latency (MWT) ≥20 min across dose groups was as follows: 37% in the 0.5 mg bid group, 81% in the 2 mg bid group, 81% in the 2 mg → 5 mg qd group, and 61% in the 7 mg qd group [95,96]. To date, the OX2R agonist TAK-861 has shown no hepatotoxicity in trials, with adverse events primarily mild to moderate (e.g., controllable insomnia, urinary urgency, and frequency), suggesting strong potential for clinical application. However, current clinical studies of OX2R agonists lack head-to-head comparisons with comparable agents. Future long-term extension trials are needed to further validate its sustained efficacy and long-term safety. Given the history of TAK-994, rigorous liver safety monitoring remains critical throughout this drug class’s development and any future post-marketing phase. If approved, a Risk Evaluation and Mitigation Strategy (REMS) program, including baseline and periodic liver enzyme tests, would be a prudent risk mitigation measure.

In studies of exogenous orexin replacement therapy, animal experiments demonstrate that orexin supplementation can reverse all core symptoms of narcolepsy type 1 (NT1). However, human studies reveal that while exogenous orexin-A improves sleep structure, olfactory function, and attention in NT1 patients, it fails to significantly alleviate excessive daytime sleepiness—the core clinical manifestation. This discrepancy suggests that orexin deficiency may not be the sole mechanism underlying NT1 pathogenesis. Other potential pathological mechanisms include autoimmune responses damaging orexin neurons while also impairing their receptors; dysregulation of related neural networks within the hypothalamus, such as concomitant histaminergic pathway dysfunction; or loss of neural pathway plasticity due to chronic orexin deficiency, rendering exogenous orexin ineffective at activating arousal-related circuits.

Future therapeutic strategies for narcolepsy type 1 (NT1) targeting the orexin system should prioritize the following directions. First, accelerating the clinical development of OX2R agonists, including head-to-head drug comparisons and evaluations of long-term efficacy, safety, and real-world effectiveness. Second, addressing key evidence gaps by clarifying the clinical benefits of exogenous orexin-A in alleviating non-core symptoms such as cataplexy and sleep paralysis. Third, systematically refining intervention protocols through comparative studies of orexin dosage, frequency, and administration routes (e.g., intranasal, intrathecal) to establish reliable dose-response relationships. Finally, developing multi-target combination therapies—such as pairing selective OX2R agonists with conventional arousal-promoting agents or combining exogenous orexin with receptor agonists—may address the complex pathophysiology of NT1 and offer novel solutions for refractory or severe cases. With ongoing clinical trials and continuous optimization, orexin-targeted therapies are poised to fundamentally reshape NT1 treatment.

Current immunotherapy approaches targeting NT1 primarily focus on B-cell-targeted interventions or broad-spectrum immunosuppression. However, recent immunological studies reveal that CD8+ T cells are the primary effector cells responsible for the selective destruction of orexinergic neurons. Consequently, future immunotherapy development should prioritize the creation of drugs targeting CD8+ T cells (such as alimertinib and natuzumab), with their efficacy validated through rigorous clinical trials. Regarding the optimal intervention timing, clinical observational data indicate that approximately 80% of orexin neurons have already undergone irreversible loss by the time cataplexy symptoms appear. This strongly suggests that effective mechanism-based treatments must be initiated prior to symptom onset. Specifically, the intervention window should be set after the emergence of excessive daytime sleepiness but before the occurrence of cataplexy. For example, in high-risk populations such as those positive for HLA-DQB1*06:02, dynamic monitoring of cerebrospinal fluid orexin levels and CD8+ T cell-related markers could serve as biological indicators for initiating early intervention. Regarding efficacy assessment systems, current approaches suffer from insufficient standardization. Existing assessment tools are heterogeneous, encompassing subjective scales (e.g., Epworth Sleepiness Scale, ESS) and objective measures (e.g., Multiple Sleep Latency Test, MSLT; CSF orexin testing). Furthermore, symptom presentation in pediatric patients differs significantly from adults, necessitating the development of age-specific standardized assessment tools (e.g., child-specific cataplexy diaries) to enhance accuracy and sensitivity. Notably, existing evidence on immunotherapy primarily stems from case reports or non-randomized controlled studies, whose results are susceptible to confounding factors like placebo effects and natural disease fluctuations (some patients may experience short-term spontaneous remission). Therefore, rigorously designed and executed high-quality randomized controlled trials are essential to confirm the true efficacy and safety of immunotherapy. Future research and clinical practice in NT1 immunotherapy should prioritize three key directions: developing CD8+ T cell-targeted therapeutics, optimizing early intervention strategies during prodromal phases, and establishing age-specific standardized efficacy assessment systems. Only through systematic advancement in these areas can the precise role and clinical value of immunotherapy within the overall NT1 management strategy be definitively established.

Cell and gene therapies represent a long-term vision for fundamentally restoring the function of the orexinergic system. Cell therapy involves transplanting stem cell-derived orexinergic neuronal precursors into the brain. Current challenges include (a) limited donor cell sources, still relying largely on postnatal animal hypothalamic tissue; (b) extremely low post-transplantation cell survival rates, reported to be only about 5%; and (c) insufficient evidence of functional integration with host circuits or symptomatic improvement. Future progress depends on generating fully functional orexin neuroblasts that exhibit (1) enhanced survival after transplantation; (2) physiologically regulated orexin release; (3) molecular, morphological, and electrophysiological properties resembling mature orexin neurons; (4) capacity to form functional orexin-releasing terminal networks; and (5) successful integration into host neural circuits to restore arousal regulation. Achieving these goals requires systematic preclinical research using robust animal models.

Gene therapy has demonstrated significant efficacy in multiple narcolepsy mouse models, with effects varying by targeted brain region. Key areas such as the ZI, LH, dorsolateral pons, and amygdala regulate cataplexy, wake maintenance, and circadian rhythms via distinct pathways. These findings support the preclinical foundation for precise gene therapy in human narcolepsy. However, as human narcolepsy is primarily acquired—unlike the genetic orexin-KO models used—validation in more clinically relevant models (e.g., autoimmune orexin neuron injury) is necessary. This field is still in the preclinical research stage and has some way to go before clinical application.

The evaluation of non-pharmacological interventions and overall treatment success in narcolepsy would benefit from standardizing patient-reported outcomes (PROs). Current PRO measures vary widely, limiting comparability. We recommend that future trials and observational studies adopt a core PRO set, minimally including the Epworth Sleepiness Scale (ESS), Functional Outcomes of Sleep Questionnaire (FOSQ), EQ-5D-5L, and a validated depression scale (e.g., PHQ-9).

Several limitations of this study should be noted. First, as a narrative rather than a systematic review, it may be subject to selection bias. Moreover, the quality of included studies—including randomized trials and real-world evidence—was not formally evaluated using standardized risk-of-bias tools (e.g., Cochrane RoB 2). Thus, our conclusions are conditional, and potential bias in the primary literature must be acknowledged. Second, direct head-to-head comparisons among many evaluated agents—particularly newer OX2R agonists versus established therapies—are lacking. To this end, we have summarized the design, potential biases, and methodological limitations of key randomized controlled trials (RCTs) and real-world studies evaluating major pharmacological interventions for NT1 in Table 8. Additionally, the underlying assumptions of cited network meta-analyses (e.g., transitivity and consistency) were not examined, and their findings should be interpreted cautiously. Third, robust long-term safety and efficacy data in special populations (e.g., pediatric and geriatric patients) remain scarce, warranting further study. Finally, the shift toward pathophysiology-targeted therapies like OX2R agonists and LXB raises health economic concerns. The potentially high cost of these novel agents, alongside limited pediatric access and reimbursement challenges, may exacerbate treatment inequities. Formal cost-effectiveness analyses comparing new and existing treatments are urgently needed to inform policy and ensure equitable access, especially for children and resource-limited settings.

## 5. Conclusions

Current NT1 management relies on pharmacological and non-pharmacological strategies for symptom control. First-line EDS pharmacotherapy includes wake-promoting agents (e.g., modafinil, solriamfetol and pitolisant), while sodium oxybate and its derivatives are central to cataplexy management and also improve nocturnal sleep. Non-pharmacological interventions like scheduled napping and CBT provide essential adjunctive support.

The treatment paradigm is shifting towards mechanism-based therapies. The OX2R agonist TAK-861 shows exceptional promise, demonstrating robust efficacy in improving EDS and cataplexy in Phase III trials, with the potential to become a first-line therapy. While challenges remain for other emerging approaches like immunotherapy and orexin replacement, OX2R agonists represent a transformative advance. Future management will increasingly focus on personalized, pathophysiology-targeted treatments.

## Figures and Tables

**Table 1:** Summary of Guideline Recommendations for Pharmacological Treatment of Narcolepsy in Adults.

Symptom	Treatment	AASM (2021)	EAN/ESRS (2021)
EDS	Modafinil	Strong	Strong
	Armodafinil	Conditional	Weak
	Pitolisant	Strong	Strong
	Sodium Oxybate	Strong	Strong
	Solriamfetol	Strong	Strong
	Methylphenidate	Conditional	Weak
	Dextroamphetamine/Amphetamine derivatives	Conditional	Weak
Cataplexy	Sodium Oxybate	Strong	Strong
	Pitolisant	Strong	Weak
	Antidepressants (e.g., Venlafaxine, Clomipramine, Fluoxetine, Citalopram)	Not recommended	Strong
DNS	Sodium Oxybate	Not mentioned	Strong
SP/HH	Pitolisant	Not mentioned	Weak
	Sodium Oxybate	Not mentioned	Weak
	Antidepressants *	Not mentioned	Weak

Abbreviations: AASM, American Academy of Sleep Medicine; EAN/ESRS, European Academy of Neurology/European Sleep Research Society; FDA, U.S. Food and Drug Administration; EMA, European Medicines Agency; EDS, excessive daytime sleepiness; DNS, disturbed nocturnal sleep; SP, sleep paralysis; HP/HH, hypnagogic/hypnopompic hallucinations. * Antidepressants include SSRIs, SNRIs, and TCAs.

**Table 2:** Summary of Guideline Recommendations for Pharmacological Treatment of Narcolepsy in Children.

Symptom	Treatment	AASM2021	EAN/ESRS2021
EDS	Modafinil	Conditional	Weak
	Sodium Oxybate	Conditional	Strong
	Pitolisant	Not recommended	Weak
	Methylphenidate	Not recommended	Weak
	Dextroamphetamine	Not recommended	Weak
Cataplexy	Sodium Oxybate	Conditional	Strong
	Pitolisant	Not recommended	Weak
	Antidepressants *	Not recommended	Weak
DNS	Sodium Oxybate	Not mentioned	Weak
SP/HH	Sodium Oxybate	Not mentioned	Weak
	Antidepressants *	Not mentioned	Weak

Abbreviations: AASM, American Academy of Sleep Medicine; EAN/ESRS, European Academy of Neurology/European Sleep Research Society; FDA, U.S. Food and Drug Administration; EMA, European Medicines Agency; EDS, excessive daytime sleepiness; DNS, disturbed nocturnal sleep; SP, sleep paralysis; HP/HH, hypnagogic/hypnopompic hallucinations; * Antidepressants include SSRIs, SNRIs, and TCAs.

**Table 3:** Pharmacology of Pharmacological Treatments for Narcolepsy in Adults.

Drug	Daily Dosage Range	Dosing Schedule	Tmax (h)	t½ (h)	Mechanism of Action	Abuse Potential	Regulatory Status
Modafinil	100–400 mg	Initial: 100–200 mg once daily; may increase weekly by 100 mg up to 400 mg, once or divided	2–4	15	Weak DAT inhibitor	Low	FDA: Approved; EMA: Approved
Armodafinil	100–250 mg	Initial: 50–150 mg once daily; may increase weekly by 50 mg up to 250 mg, once or divided	2	~15	Weak DAT inhibitor	Low	FDA: Approved EMA: No
Pitolisant	9–36 mg	Initial: 8.9 mg daily for 1 week, then 17.8 mg; max 35.6 mg; CYP2D6 poor metabolizers: half max dose	3.5	~20	H3-receptor antagonist/inverse agonist	Low	FDA: Approved EMA: No
Sodium Oxybate (IR)	4.5–9 g/night (containing 820–1640 mg sodium)	Initiate dosage at 4.5 g/night orally, divided into two doses (at bedtime and 2.5 to 4 h later); titrate 1.5 g per night at weekly intervals; Recommended dosage: 6 g to 9 g/night.	0.5–1.25	0.5–1	GABA modulator	High	FDA: Approved EMA: Approved
Low-Sodium Oxybate	4.5–9 g/night (containing 87–131 mg sodium)	Initiate dosage at 4.5 g/night orally, divided into two doses (at bedtime and 2.5 to 4 h later); titrate 1.5 g per night at weekly intervals; Recommended dosage: 6 g to 9 g/night.	1.3	0.67	GABA modulator	High	FDA: Approved EMA: No
Once-Nightly Oxybate	4.5–9 g/night (containing 820-1640mg sodium)	Initiate dosage at 4.5 g once per night orally; titrate to effect in increments of 1.5 g per night at weekly intervals; Recommended dosage range: 6 g to 9 g once/night orally.	1.5	0.5–1	GABA modulator	High	FDA: Approved EMA: No
Solriamfetol	75–150 mg	Initial: 75 mg once daily; may increase every ≥3 days to 150 mg	2	~7.1	DAT and NET inhibitor	Moderate	FDA: Approved EMA: No
Methylphenidate	10–60 mg	Initial: 10 mg twice daily; may increase weekly by 5–10 mg; max 60 mg in 2–3 divided doses	1–14	2–7	DAT inhibitor	Moderate–High	FDA: Approved EMA: Approved
Dextroamphetamine	5–60 mg	Initial: 10 mg once daily; may increase weekly by 10 mg; max 60 mg once daily or divided	IR: 3, ER: 8	~12	DAT and NET inhibitor	High	FDA: No EMA: Approved

Notes: Abbreviations: Tmax: Time to maximum concentration; t½: Elimination half-life; DAT: Dopamine transporter; NET: Norepinephrine transporter; GABA: γ-aminobutyric acid; IR: Immediate-Release formulation; ER: Extended-Release formulation. Dosing schedules represent general guidelines; individual titration based on efficacy and tolerability is recommended; Abuse potential categories: Low = minimal abuse potential; Moderate = some abuse potential; High = significant abuse potential; Sodium content varies among oxybate formulations: IR solution (410–1640 mg sodium), Low-sodium (87–131 mg sodium), Once-nightly (510–1120 mg sodium); CYP2D6 poor metabolizers require dose adjustment for pitolisant.

**Table 4:** Pharmacology of Pharmacological Treatments for Narcolepsy in Children.

Drug	Daily Dosage Range	Dosing Schedule (Oral)	Tmax (h)	t½ (h)	Mechanism of Action	Abuse Potential	Regulatory Status
Modafinil	50–400 mg	<30 kg: Start 100 mg daily, max 300 mg; ≥30 kg: Start 100 mg daily, max 400 mg; may divide doses	2~4 *	15 *	Weak DAT inhibitor	Low	FDA: No EMA: No
Sodium Oxybate (IR)	2–9 g/night, based on body weight	Pediatric patients 7 years and older weighing at least 20 kg. The recommended starting dosage, titration regimen, and maximum total nightly dosage are based on body weight, divided into two doses (at bedtime and 2.5 to 4 h later).	0.5–1.25 *	0.5–1 *	GABA modulator	High	FDA: ≥7yr †; EMA: No
Low-Sodium Oxybate	2–9 g/night, based on body weight.	Pediatric patients 7 years and older weighing at least 20 kg. The recommended starting dosage, titration regimen, and maximum total nightly dosage are based on body weight, divided into two doses (at bedtime and 2.5 to 4 h later).	1.3 *	0.67 *	GABA modulator	High	FDA: ≥7yr †; EMA: No
Once-Nightly Oxybate	4.5–9 g/ night	Pediatric patients 7 years and older weighing at least 45 kg. The recommended starting dosage is 4.5 g/night. Increase the dosage by 1.5 g/night at weekly intervals to the maximum recommended dosage of 9 g/night orally.	1.5 *	0.5–1 *	GABA modulator	High	FDA: ≥7yr †; EMA: No
Pitolisant	4.5–36 mg	25~40 kg: start 4.45 mg, max 17.8 mg; ≥40 kg: start 4.45 mg, max 35.6 mg; titrate over 3–4 weeks; CYP2D6 poor metabolizers: half max dose	3.5 *	~20 *	H3-receptor antagonist/inverse agonist	Low	FDA: ≥6yr; EMA: No
Methylphenidate	10–40 mg (IR/ER)	Start 5 mg twice daily; max 60 mg/day; ER may replace IR once dose stabilized	1–14	2–7	DAT inhibitor	Moderate–High	FDA:No; EMA: No
Dextroamphetamine	2.5–20 mg (divided)	Children 6–12 years: start 5 mg daily; max 60 mg; Adolescents: start 10 mg daily; max 60 mg; IR or ER formulations	IR: 3, ER: 8	~12 *	DAT and NET inhibitor	High	FDA: No; EMA: No

Notes: Abbreviations: Tmax: Time to maximum concentration; t½: Elimination half-life; DAT: Dopamine transporter; NET: Norepinephrine transporter; GABA: γ-aminobutyric acid; IR: Immediate-Release formulation; ER: Extended-Release formulation. Pediatric dosing is weight-based and age-dependent; doses should be individualized; * indicates pharmacokinetic parameters extrapolated from adult studies due to limited pediatric data; Most medications are used off-label for pediatric narcolepsy treatment; † Sodium oxybate formulations approved for narcolepsy in children ≥7 years in the US; Methylphenidate and dextroamphetamine have FDA approval for ADHD but not for narcolepsy in the pediatric population; CYP2D6 genotyping may be considered for pitolisant dosing optimization.

**Table 5:** Non-pharmacological Treatment Interventions for Narcolepsy and Their Target Effects.

Intervention Category	Specific Measures	Target Outcomes
Behavioral Therapy (EDS)	Scheduled short naps (15–20 min/nap, 2–3 times/day); Sleep extension therapy	Reduce EDS; improve daytime alertness
Cognitive Behavioral Therapy	Cognitive restructuring; Systematic desensitization (for cataplexy); Problem-solving	Improve functional cognition; manage emotional triggers; enhance treatment adherence
Physical Exercise	Daily regular exercise	Reduce EDS; regulate sleep–wake cycle; control body weight
Counseling	Patient education; Peer support	Improve symptom management skills; build confidence in coping with the disease
Family Support	Caregiver education; Parent-child support	Help caregivers manage the patient’s condition; alleviate psychological distress

**Table 6:** List of clinical trials of OX2R Agonist for narcolepsy treatment.

Drug Name	Trial Phase	Trial ID	Date of Registration	Recruitment Status
TAK-861	3	CTIS2024-511998-30-00	13 June 2024	Not Recruiting
	3	CTIS2023-508465-32-00	25 April 2024	Not Recruiting
	2	EUCTR2022-002966-34-FI	20 January 2023	Not Recruiting
	2	EUCTR2022-002966-34-IT	14 December 2022	Not Recruiting
	2	NL-OMON53780	12 December 2022	Not Recruiting
	2	EUCTR2022-002966-34-SE	5 December 2022	Not Recruiting
	2	EUCTR2022-001654-38-NL	5 December 2022	Authorised
	2	NL-OMON53459	5 December 2022	Recruiting
	2	EUCTR2022-002966-34-NO	5 December 2022	Not Recruiting
	2	EUCTR2022-001654-38-SE	18 November 2022	Not Recruiting
	2	EUCTR2022-001654-38-FI	16 November 2022	Not Recruiting
	2	EUCTR2022-001654-38-NO	28 October 2022	Not Recruiting
	2	EUCTR2022-001654-38-FR	21 October 2022	Not Recruiting
	2	JPRN-jRCT2071210007	8 April 2022	Not Recruiting
TAK-994	2	JPRN-jRCT2071210015	28 April 2021	Not Recruiting
	2	NCT04820842	26 March 2021	Not recruiting
	2	JPRN-jRCT2080225083	21 February 2020	Not Recruiting
	2	NCT04096560	18 September 2019	Not recruiting
ALKS 2680	2/3	NCT06767683	6 January 2025	Recruiting
	2	NCT06555783	13 August 2024	Recruiting
	2	NCT06358950	2 April 2024	Not recruiting
ORX750	2	NCT07096674	2 July 2025	Recruiting
	2	NCT06752668	23 December 2024	Recruiting
TAK-360	2	JPRN-jRCT2051250080	31 July 2025	Recruiting
	2	NCT06952699	23 April 2025	Recruiting
E2086	1	NCT06462404	12 June 2024	Not recruiting

**Table 7:** Summary of Immunotherapy in NT1.

Treatment Category	Representative Solution/Medication	Mechanism	Effect of Typical Case Studies	Key Limitations
Multi-effect Immunotherapy	Corticosteroids (Prednisone, IVMP)	Inhibits the synthesis of inflammatory mediators and impairs the function of neutrophils, monocytes, and B/T cells.	8-year-old male (onset 2 months ago): Prednisone 1 mg/kg/day for 3 weeks, with no improvement in EDS or sleep parameters [120];	Effects were inconsistent, with mostly short-term improvements. CSF orexin-A levels remain unchanged. Long-term use carries numerous side effects (such as acne and dermatitis).
			2 adults with concomitant inflammatory conditions (inflammatory bowel disease, asthma): 40mg/day prednisone resulted in resolution of EDS and cataplexy (likely related to the central stimulatory effects of corticosteroids) [121].	
	Plasma Exchange (PLEX)	Clear circulating antibodies and cytokines	Only 1 case: 60-year-old female (onset 2 months prior): Symptoms improved by 80% after 5 days of PLEX treatment, but relapsed 3 days later; subsequent IVIG treatment was ineffective [122].	The effect is short-lived with no long-term benefits. Only one case has been reported, rendering it of no value for broader application.
	Intravenous Immunoglobulin (IVIG)	Regulate immune balance and suppress autoimmune responses	Early intervention (within 1–4 months of onset): Some patients experience reduced drop attack frequency and improved EDS, though effects typically last only weeks to months [123];	Heterogeneous efficacy (significant differences between children and adults), lack of randomized controlled trials, inability to confirm definitive therapeutic efficacy
			Non-randomized study (22 patients receiving IVIG vs. 30 patients receiving standard treatment): No significant difference observed, with evidence of a placebo effect (patients in the double-blind trial reported improvement with both IVIG and placebo) [124].	
Targeted B/T Cell Therapy	Rituximab (anti-CD20, B-cell depletion)	Deplete mature B cells and suppress humoral immunity	1 case of Post-Pandemrix NT1 with psychiatric symptoms: Symptoms improved in the short term (2 months), subsequent infusions were ineffective [125]; 1 case of a 28-year-old male: 5 treatments of 1000mg/6 months, subjective improvement in EDS but no change in syncope, CSF orexin-A progressively decreased [126].	Irreversible neuronal loss, no sustained benefit after B-cell depletion, potential risk of infection
	Alemtuzumab (anti-CD52, T-cell suppression)	Inhibiting CD4+ T cells may exert neuroprotective effects.	1 Case: 79-year-old male (62-year history of NT1): cataplexy completely resolved during treatment; no change in other symptoms [127].	Only 1 case reported, mechanism unclear (may involve neuroprotection rather than immunosuppression), lacks large-scale validation.
	Nalizumab (anti-α4 integrin)	Prevent T cells from entering the central nervous system	1 case of a 21-year-old female (onset 3 months ago): Symptoms showed no improvement after IVIG treatment; CSF orexin-A decreased from 70 pg/mL to 17 pg/mL [128].	Early intervention remains ineffective, likely due to extensive neuronal loss (neural cell loss reaches 80% by the onset of syncope), with a risk of progressive multifocal leukoencephalopathy (PML).

**Table 8:** Summary of Key RCTs and Real-World Studies on New Drugs for Narcolepsy: Design, Potential Biases, and Methodological Limitations.

Study	Trial Design	Potential Biases	Methodological Limitations
OX2R agonists, TAK-994, Dauvilliers Y, 2023 [93]	Phase 2, RCT, double-blind, placebo-controlled, dose-finding; multicenter; NT1; 8-week treatment	1. Early termination leading to incomplete data 2. Lack of independent external monitoring board 3. Risk of unblinding due to AEs	1. Small sample size with high dropout rate 2. Short duration; long-term safety/efficacy unknown 3. No active comparator; limited generalizability to NT1
OX2R agonists, TAK-861, Dauvilliers Y, 2025 [95]	Phase 2, RCT, double-blind, placebo-controlled; international multicenter; NT1; 8-week treatment	1. Functional unblinding due to efficacy/side effects 2. Selection bias from liver disease exclusion 3. Recall bias in self-reported outcomes	1. Small sample size (*n* = 112); limited subgroup power 2. Short duration; long-term data lacking 3. No active comparator; high dropout in assessments
OX2R agonists, The First Light Study, 2025 [96,97]	Phase 3, RCT, double-blind, placebo-controlled; NT1; 12-week treatment + 4-week follow-up	1. Selection bias due to HLA-DQB1*06:02 inclusion 2. Performance bias from TEAEs leading to unblinding 3. Detection bias in subjective endpoints	1. Small sample size (*n* ≈ 167); reduced statistical power 2. Short duration; long-term assessment lacking 3. Limited global diversity in recruitment
OX2R agonists, The Radiant Light Study, 2025 [96,97]	Phase 3, RCT, double-blind, placebo-controlled; NT1 with cataplexy; 12-week treatment + 4-week follow-up	1. Selection bias due to strict inclusion criteria 2. Attrition bias from missing dropout data 3. Reporting bias in patient-reported outcomes	1. Limited dose comparison (single active dose) 2. Short follow-up; long-term AEs not assessed 3. Reliance on conventional endpoints; lack of novel measures
Pitolisant Harmony III, Dauvilliers Y, 2019 [23]	Phase 3, open-label, single-arm, pragmatic; long-term; previously exposed patients	1. Lack of blinding introducing performance/detection bias 2. Selection bias from prior exposure 3. High dropout due to perceived inefficacy	1. No placebo/active comparator; causal inference limited 2. No objective sleep measures 3. Concomitant medications confound outcomes
Pitolisant Pediatric, Dauvilliers Y, 2023 [26]	Phase 3, RCT, double-blind, placebo-controlled; children aged 6–17 years; NT1/NT2	1. Unblinding due to discernible AEs 2. Underestimated effect from concomitant medications 3. Recall bias in cataplexy reporting	1. Short duration (8 weeks); long-term efficacy unknown 2. UNS endpoint not validated in pediatrics 3. Underpowered subgroup analyses
Pitolisant Real-World Interim, Giuseppe Plazzi, 2025 [25]	Prospective, non-interventional PASS; multicenter; Europe; long-term follow-up	1. Selection bias from specialized centers 2. Reporting bias from patient/physician reports 3. Confounding by concomitant treatments	1. No control/comparator group 2. High discontinuation/loss to follow-up 3. Unblinded design with observer bias
Solriamfetol Phase 3, Michael J, 2019 [32]	Phase 3, RCT, double-blind, placebo-controlled; NT1/NT2; EDS; 12-week treatment	1. High discontinuation in 300 mg group affecting interpretability 2. Prior stimulant use affecting baseline 3. Unblinding due to AEs	1. Short duration; long-term data lacking 2. Not powered for cataplexy assessment 3. No active comparator; indirect comparisons
Solriamfetol Long-term, Malhotra, 2020 [35]	Phase 3, open-label extension with randomized withdrawal; narcolepsy/OSA; up to 52 weeks	1. Open-label design introducing bias 2. Selection bias from prior trial completers 3. Unblinding during withdrawal phase	1. No active comparator; comparative efficacy limited 2. Reliance on subjective measures without objective tests 3. Generalizability limited by exclusions
Solriamfetol SURWEY, Y Winter, 2023 [138]	Retrospective, non-interventional chart review; Germany; real-world; stratified subgroups	1. Selection bias from stable dose completion 2. Reporting bias from unblinded outcomes 3. Recall bias from medical records	1. Small sample size (*n* = 70); limited generalizability 2. No control group; causal inference limited 3. Variable follow-up and titration schedules
Sodium Oxybate Pediatric, Lecendreux, 2022 [48]	Phase 3, RCT, double-blind, randomized withdrawal; pediatric (7–16 years); NT1 with cataplexy	1. Unblinding due to drug effects 2. Funding bias from sponsor 3. Reliance on subjective outcomes	1. Small sample in younger age group 2. Open-label extension lacks placebo control 3. Post hoc analyses without multiplicity adjustment
Low-Sodium Oxybate (LXB) Phase 3, Bogan, 2021 [139]	Phase 3, RCT, double-blind, placebo-controlled withdrawal; adults; NT1 with cataplexy	1. Selection bias from responder enrichment 2. Unblinding due to AEs differences 3. Withdrawal effects confounding outcomes	1. No direct comparison with SXB 2. Short withdrawal period (2 weeks) 3. Limited cardiovascular assessment
Once-Nightly SXB (FT218) Phase 3, Kushida, 2022 [140]	Phase 3, RCT, double-blind, placebo-controlled; NT1/NT2; multiple doses; 13-week treatment	1. Attrition bias from higher discontinuation in active group 2. Unblinding due to known AEs 3. Concomitant stimulant use influencing EDS	1. Capped cataplexy reporting underestimating effect 2. Placebo effect diluting treatment effect 3. Short duration; long-term data lacking
Low-Sodium Oxybate (LXB) Long-term, Bogan, 2023 [52]	Phase 3, RCT, double-blind, placebo-controlled withdrawal; NT1; open-label extension	1. Expectation bias from open-label periods 2. Attrition bias from differential dropout 3. Recall bias in AE reporting	1. Open-label design affecting subjective outcomes 2. Small subgroup samples limiting power 3. No systematic AE timing assessment

Notes: AEs: Adverse events; EDS: Excessive daytime sleepiness; ESS: Epworth Sleepiness Scale; HLA: Human leukocyte antigen; IT: Intention-to-treat; MWT: Maintenance of Wakefulness Test; NT1: Narcolepsy type 1; NT2: Narcolepsy type 2; OLOTTP: Open-label optimization and titration period; OLE: Open-label extension; OSA: Obstructive sleep apnea; PASS: Post-authorization safety study; PGI-C: Patient Global Impression of Change; RCT: Randomized controlled trial; SXB: Sodium oxybate; TEAEs: Treatment-emergent adverse events; UNS: Ullanlinna Narcolepsy Scale. Trials are categorized by drug class. Design and population summaries are simplified for clarity. Potential biases and limitations highlight key issues as reported in the original documents.

## Data Availability

No new data were created or analyzed in this study.
